# Brief Account of the Present State of Medical and Physical Literature in Italy

**Published:** 1803-05-01

**Authors:** 


### Brief Account *of the present* State *of* Medical *and* Physical Literature *in* Italy; *communicated by Dr.* Noehden.

Dr. Nolde, professor of the university of Rostock, in the dutchy of Meklenbourg, has lately made a literary tour through different parts of Italy, of which he gives the following account in a letter addressed to the Editor of the Salzburg Medical and Chirurgical Gazette. Of all the countries that have experienced the dreadful afflictions of the late war, none seems to have suffered so much as Italy, a country that will for a long time feel the consequences of those ravages by which it was devastated, and still bleeds of the wounds that were inflicted on the morality and industry of its inhabitants, and to the sciences with which it had long flourished. The study of medicine was equally neglected, and the medical schools, so much celebrated, were disturbed and dispersed. The hospitals were loaded with taxes and contributions, their property seized, their subsistence diminished; and at a time when poverty and diseases should have made them the refuge of want and misery, the number of patients which could have been received were unmercifully restrained.

About this time, when the revolutionary spirit had conquered the minds of the people, the Brunonian system became known amongst the Italian physicians, and their minds being filled with revolutionary ideas, it is easily conceived how ardently a doctrine would be received which threatens to subvert the hitherto known dogmas of medicine, even when sanctioned by the authority of ages. It cannot be denied, that the too precipitate and inconsiderate use or practical application of that doctrine has been extremely prejudicial to the life and health of many. Professor N. met with several practitioners in Italy, who though being themselves well wishers to the Brunonian system, openly confessed how detrimental the injudicial application of its principles at the sick bed had generally proved, and that it was rather to be ascribed to a lucky accident, if such a treatment had ever been attended with success. At the present time, however, the revolutionary enthusiasm having nearly subsided, the Italian physicians have begun to examine that system with a more unprejudiced mind, to expose it to the test of experience and observation, and to employ it with greater restriction. Zealous Brunonians are are not now so frequently to be met with as before. Amongst the rational physicians of upper Italy, Dr. Rasori, formerly professor of the practice of physic at the university of Pavia, deserves particularly to be mentioned. This gentleman, who is favourably known to the public by several publications, and especially by his elaborate description of a very fatal epidemic fever at Genoa, has acknowledged, and pointed out the errors of Brown's system; in lieu of which he endeavours to establish a new one, namely, the system of the contra stimuli. Being at present engaged in translating Darwin's Zoonomia into the Italian language, he has deferred publishing his system till this translation has made its appearance, and to that period he defers its discovery. It is only known, that he was led to this new theory of medicine by the thus called depressing passions, and that he utterly denies the indirect debility of Dr. Brown. He assures his friends, that he has proved his theory by experiments made on animals as well as by observations at the sick bed. When it shall have been made public, professor Borda, of Pavia, intends publishing a Materia Medica adapted to the principles of this new system. The state of Medicine in the Roman and Neopolitan dominions is not very promising; there are few countries, in which the generality of medical men have kept so little pace with the modern improvements in the healing art; and it were rather to be wished, that the Brunonian theory had made some progress amongst them, in order to arouse them from their state of supineness.

The remedy of Dr. Reich in fevers has not been favourably received in Italy, except by Dr. Lusi and the two Flajanis at Rome; young Flajani has translated Dr. Reich's publication, to which he has added some observations of his own.

Of all the new discoveries lately made in the medical art, the cow-pox inoculation has particularly engaged the attention of the Italian physicians; the most important observations and experiments, however, relative to this great object have been made in the Italian republic, where, it is said, that above seventy thousand individuals have been inoculated, of which Dr. Sacco has vaccinated in the Milanese about twenty thousand. The work, in which he has published his observations, is sufficiently known to our readers. Still more interesting than Dr. Sacco's work, seems to be the following publication, which appeared by order of the committee appointed for the purpose of investigating the use of the cow-pox inoculation. " *Risul-*

*tati*

*tati di osservazioni e sperienze sull' Inoculazione del Vajuolo Vaccino, institut. nello spedale maggiore di Milano dalla commissione medico-chirurgica superiormente de legata a quest oggetto. Milan.* 1802." Professor Fanzago in Padoua, and some other physicians in upper Italy, have likewise written upon this subject.

At Naples, where the small-pox have always been extremely fatal, Dr. Marshall, an Englishman, has the merit of having introduced the cow-pox inoculation, for which purpose he has published a small popular work, entitled, *Osservazione sopra il Vajuola Vaccino di Giuseppe, Marshall, M. D. Palermo,* 1801. The first child inoculated for the cow-pox was, by order of the king of Naples, re-inoculated with the small-pox, which however did not take, though this inoculation was once more repeated. From that time the cow-pox inoculation has met with a favourable reception, and begins to make much progress in the Neopolitan dominions. In Florence however, Siena, Pisa, and in the whole dutchy of Toscana, this great gift of heaven is almost generally disregarded, and a general prejudice has arose against the introduction of the vaccine inoculation, which some unfortunate cases seem to have produced, in which children happened to be inoculated for the cow-pox, who being previously infected with the small-pox contagion, got the small-pox, and died in consequence of them.

Galvanism has to this period not yet been employed in Italy to medical purposes, and no experiments have as yet been made with it on any patient. Mr. Volta lives at present in Como, his native place, retired from all public business. The antiphlogistic system of medicine is generally known and adopted; the apothecaries' shops, however, are on the whole in the most miserable state, except in upper Italy, where the medical police was always better regulated than in other parts of Italy, particularly before the time of the late war. At Rome and Naples they are bad beyond description.

The new chemical nomenclature of Signor Brugnatelli is sufficiently known. The following work has been lately published by the same author. "*Farmacopea al uso degli speciali e medici moderni della republica Italiana. Aggiuntovi la ta vola della sinonymia delle moderne nomenclature chimiche e la tarriffa delle preparazioni in questa Farmacopea, riportati da L. Brugnatelli, Pavia,* 1802." Signor Rezia, who is the present director of all the military hospitals in the Italian republic, is about to publish a Pharmacopea copoea Militaris. Signor Gallini has lately published a compendium of Physiology and Pathology, 1802, which however seems to be much inferior to his Physiological Researches. Signor Flajani, the father, has not long ago edited the third volume of his Chirurgical Observations. Signor Nannoni, in Florence, has published, "*Viaggi e opere di Lorenzo Nannoni, chirurgo in capo dell' arcispedale di Firenze, Livorno,* 1801." Another work deserves to be noticed, viz. "*Della cura Fisica e politica dell' uomo di Giovanni Pozzi. Milano, a.* x. Professor Scarpa has edited a work on the diseases of the eyes; and of the following work the first volume has made its appearance. "*Teoria della prosperita fisica delle nazione nei raporti d' economia publica, &c.* Vol. i. p. 1. *Milano,* 1801. The author of this work is Dr. Racchetti, who seems to have chiefly made use of the classic German work, published by the celebrated Dr. Frank of Vienna. Dr. Brera has again enriched the catalogue of his works with a new one, entitled, "*Lezioni medico-practiche sopra i principali vermi del corpo umano vivente e le cosi dette malattie verminosi di Valeriano Luigi Brera, Crema,* 1802. It seems to be nothing but a mere compilation. Another work is *Principi di Medicina naturale del D. Giuseppe Nicoli di Ravenna, ed.* 4. *Milano,* 1802. Dr. Francisco Vacca Berlinghieri, professor at Pisa, has published, 1801, "*La Filosofia della Medicina, al cittadino Napoleone Buonaparte.* His son, a surgeon, has edited "*Trattato dei mali venerei di Andr. Vacca Berlinghieri, publicato in Francese da P. P. Alyon, e tradotta in Italiano da Niccola Rasis di Cefalonia etc. Pisa,* 1802. The anatomical work of Caldani will be continued, and young Caldani has made a series of experiments on the structure of the bones, the results of which are contrary to those of professor Scarpa There are at present two Medical Journals publishing in Italy, one of which is conducted by Dr. Giannini in Milan; it is entitled "Memorie di Medicina," and has appeared as far as No. xvi. The second is the "Annali di Medicina," by Dr. Rasori, of which three numbers have made their appearance. The "Atti di Siena," as also the "Memorie della societa Italiana" are continued, and contain, with the Nuovo Giornale dei literati di Pisa, medical objects and treatises. *Summae plantarum a Fulgentio Vitmann in lucem editæ, anno* 1789. *Supplement.* T. i. and Instituzioni Botaniche, del Dottore Ottaviano Targioni Tozzetti, seconda edizione, Firenze, 1802, are the only botanical works to be noticed. Felice Fontana is preparing a large work on Physiology, and and Scarpa a work on aneurisms and distorted limbs. Dr. Tommassini has edited "*Lezioni critiche di Fisiologia e Pathologia, del Dott. Giacomo Tommassini,*" and Giuseppe Tortop *Instituzioni di Medicina forens.*

